# Fragility Fracture in a Young Man With Severe Depression, Malnutrition, and Reduced Mobility: A Case Report

**DOI:** 10.7759/cureus.109636

**Published:** 2026-05-25

**Authors:** Kasturi Krishnamoorthy, Clint Jomar P Bruno, Muhammad Fayyaz

**Affiliations:** 1 Geriatric Medicine, Stockport NHS Foundation Trust, Stockport, GBR

**Keywords:** bone health in male, depression, fragility fracture, osteopenia, osteoporosis in young

## Abstract

Fragility fractures in young men are uncommon and should prompt evaluation for underlying pathology. This report describes the case of a 42-year-old male patient who presented with multiple fragility fractures following a low-impact fall in the context of severe depression, alcohol misuse, and prolonged self-neglect. He was underweight (BMI 18 kg/m²), sarcopenic, functionally dependent, and had biochemical evidence of malnutrition. Management was multidisciplinary and involved orthopaedics, dietetics, and psychiatry. Following initiation of venlafaxine, there was a rapid and sustained improvement in mood, appetite, and nutritional intake. This resulted in weight gain and marked functional recovery. The development of refeeding syndrome during treatment further supported the severity of preceding malnutrition. This case highlights the importance of thorough and holistic assessment in patients with unexplained fragility fractures. Early recognition of malnutrition and its underlying drivers may prevent severe systemic complications and improve outcomes.

## Introduction

Fragility fractures typically occur in older individuals and are uncommon in younger patients, particularly in men [1]. When present, they should prompt evaluation for secondary causes of impaired bone health, including metabolic, nutritional, endocrine, and lifestyle-related factors [2].

Psychiatric conditions such as depression may contribute to reduced appetite, inadequate nutritional intake, and weight loss [3]. Malnutrition and chronic alcohol use are well-established contributors to reduced bone mineral density and increased fracture risk [4-7]. Despite this, behavioural and psychiatric factors are often under-recognized in the assessment of patients with low bone density.

We present a case of a young man with multiple fragility fractures in the context of severe depression and self-neglect. This case highlights the potential contribution of psychiatric disease to systemic physiological decline and bone fragility, and the clinical value of identifying underlying drivers early [6,8].

## Case presentation

A 42-year-old male patient presented to the emergency department following a low-impact fall from standing height after his left leg gave way while attempting to stand. He reported sacral pain and generalized weakness, with no history of high-impact trauma. He had a history of alcohol misuse, smoking, and a longstanding low mood associated with anxiety and social isolation. Over a prolonged period, he described significantly reduced oral intake due to low appetite and reduced motivation, despite no aversion to food. This resulted in progressive weight loss and functional decline. Prior to admission, he was largely wheelchair-dependent due to generalized weakness.

On examination, he appeared underweight and sarcopenic, with a body mass index of 18 kg/m². He was low in mood but cooperative. Observations were within normal limits. Trauma CT and X-ray demonstrated multiple fractures involving the spine (Figure 1), pelvis (Figure 2), and humerus (Figure 3), with radiological features of osteopenia.

**Figure 1:**
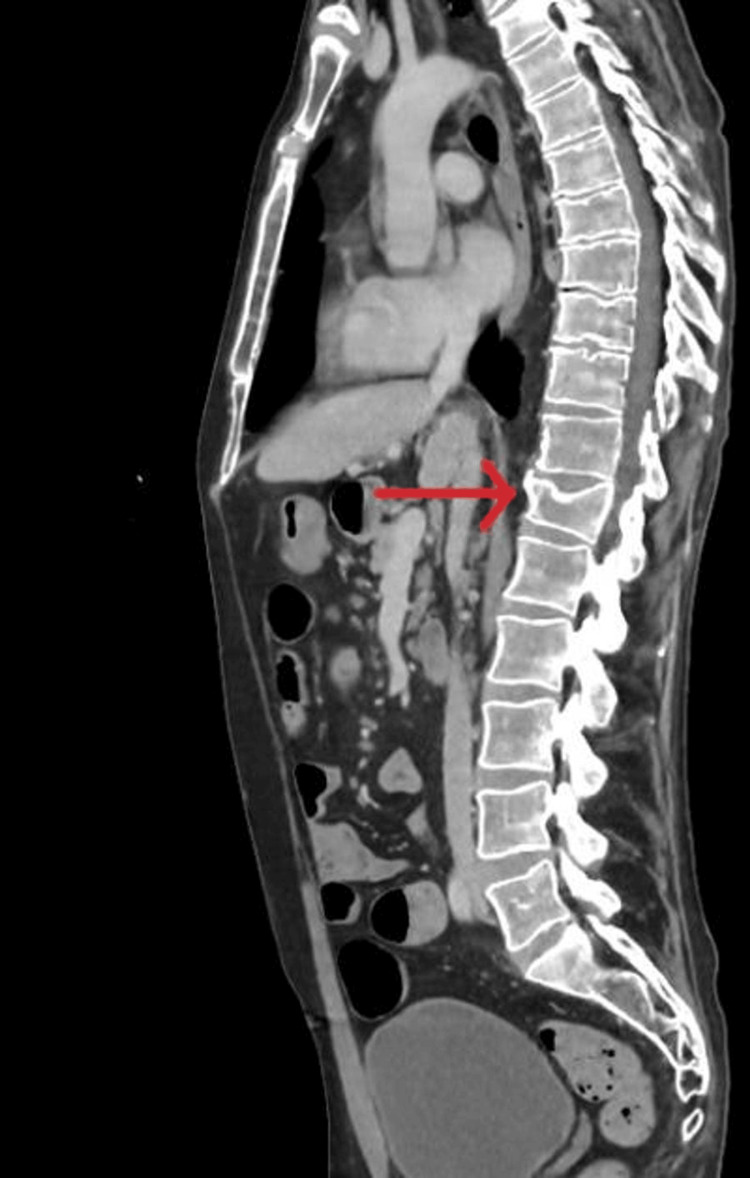
CT image of the thoracolumbar spine (sagittal view) demonstrating a fragility fracture. A compression fracture of the T12 vertebral body is seen, with features consistent with osteopenia.

**Figure 2:**
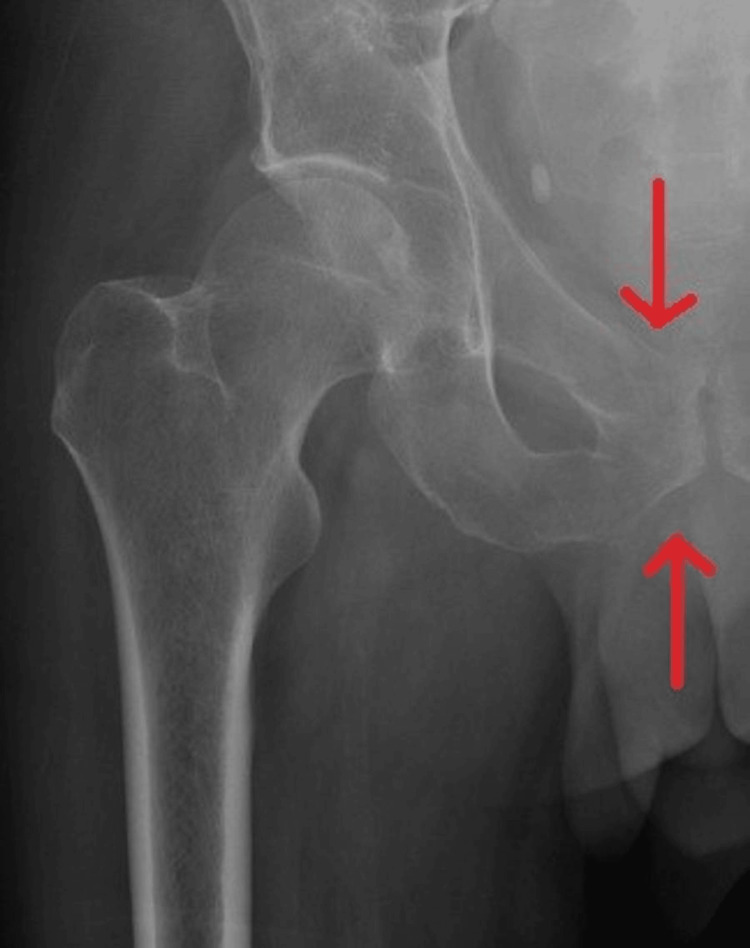
Anteroposterior X-ray of the pelvis demonstrating a pelvic fracture. A fracture of the pelvic medial ramus is seen with features of osteopenia.

**Figure 3:**
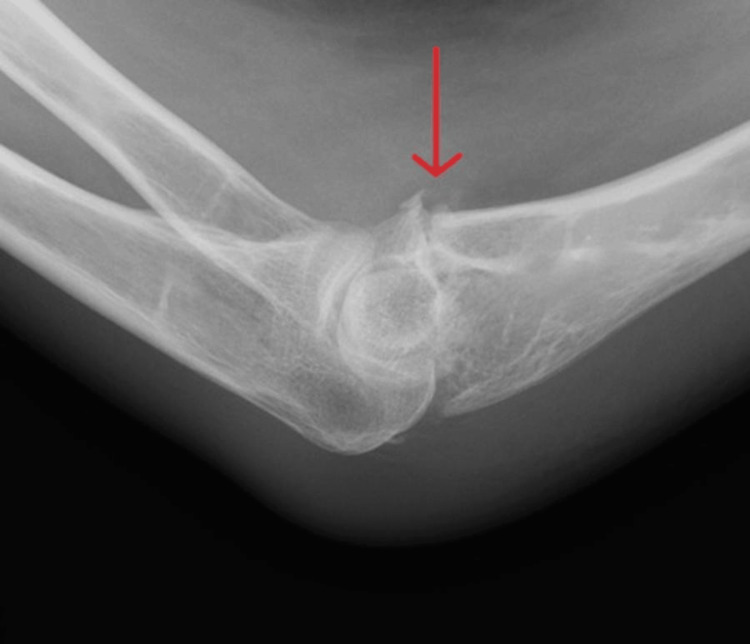
X-ray of the left humerus demonstrating a fragility fracture. A supracondylar fracture of the distal humerus is seen with features of osteopenia.

Laboratory results show low creatinine, borderline magnesium, hyponatraemia, and upper limit calcium on admission. Following initial hydration, laboratory investigations revealed anemia and electrolyte abnormalities (Table 1). Haemoglobin, magnesium, calcium, potassium, urea, and creatinine decreased during nutritional rehabilitation. However, there was a slight increase in sodium. On discharge, haemoglobin increased but remained below normal, albumin returned to 30 g/L, and sodium normalized to 138 mmol/L. Thyroid function tests showed an elevated thyroid-stimulating hormone (TSH) with normal free thyroxine on the 18th day of admission, consistent with subclinical hypothyroidism (Table 2). This may represent recovery-phase elevation of TSH in the context of systemic illness and malnutrition. 

**Table 1:** Laboratory investigations on admission, after feeding, and on discharge eGFR: estimated glomerular filtration rate

Laboratory Parameter	Reference Range	At Admission	After Feeding/Hydration	At Discharge
Hemoglobin (g/L)	130 – 180	141	95	103
Phosphate (mmol/L)	0.70 – 1.50	1.09	1.4	1.97
Protein (g/L)	60 – 80	66	44	60
Albumin (g/L)	33 – 45	30	19	30
Calcium (mmol/L)	2.20 – 2.60	2.6	2.35	2.57
Magnesium (mmol/L)	0.70 – 1.00	0.71	0.55	0.66
Sodium (mmol/L)	133 – 146	127	129	138
Potassium (mmol/L)	3.5 – 5.3	4.5	4.1	4.6
Urea (mmol/L)	2.5 – 7.8	2.6	2	2.6
Creatinine (µmol/L)	62 – 115	39	19	30
eGFR (mL/min)	–	>90	>90	>90

**Table 2:** Thyroid function TSH: thyroid stimulating hormone

Thyroid Profile	Reference Range	Patient Value
Serum Free Thyroxine (pmol/L)	10.0 – 22.1	12.9
Serum TSH (mU/L)	0.10 – 4.00	12

Nutritional assessment demonstrated weight loss from a baseline of approximately 58-53 kg on admission, with a Malnutrition Universal Screening Tool (MUST) score of 3, indicating high risk of malnutrition (Table 3). Fracture risk assessment using FRAX (Fracture Risk Assessment Tool) was above the treatment threshold, prompting referral for specialist bone health assessment.

**Table 3:** Weight, BMI, and MUST score at baseline and during admission This table shows initial weight loss compared to baseline with subsequent increase in weight and improvement in MUST score. MUST: Malnutrition Universal Screening Tool

Day	Weight (kg)	Weight change from baseline (kg)	BMI (kg/m²)	MUST Score
Pre-admission/Baseline	58	0	19.6	1
3	53.4	-4.6	18	3
11	53.8	-4.2	18.5	2
17	53.41	-4.59	18	3
24	53.7	-4.3	18.4	2
32	55.74	-2.26	19.25	1

A multidisciplinary approach was initiated. Orthopedic review recommended conservative management, and a plaster of Paris cast was applied with planned outpatient follow-up. The patient was referred to mental health services and diagnosed with features of severe depression without psychotic symptoms. Venlafaxine was commenced for these symptoms. Nutritional rehabilitation was initiated with dietetic input and oral supplementation, with close biochemical monitoring.

During nutritional rehabilitation, the patient developed features consistent with refeeding syndrome, which were managed with electrolyte replacement and monitoring. Physiotherapy and occupational therapy were involved to support gradual mobilization. Referrals were also made to rheumatology for bone health assessment and to endocrinology for evaluation of thyroid dysfunction.

Following initiation of treatment, the patient demonstrated marked clinical improvement. His mood improved, accompanied by increased appetite and oral intake. His weight increased from 53 kg to 55.7 kg, with an improvement in the MUST score from 3 to 1 (Table 3). He progressed from wheelchair dependence to independent mobilization with support, with improved strength and functional capacity. He reported a significant improvement in overall well-being compared to his pre-admission baseline and showed increased motivation to engage with ongoing care, including consideration of reducing alcohol intake and smoking.

## Discussion

This case describes an uncommon presentation of multiple fragility fractures in a relatively young man, occurring in the context of malnutrition, alcohol misuse, and depression with associated self-neglect [1]. Fragility fractures in this demographic are unusual and should prompt evaluation for secondary causes of impaired bone health. 

Malnutrition and alcohol dependence are well-recognized contributors to reduced bone mineral density [5-7]. In addition, severe depression may contribute to reduced appetite, poor nutritional intake, weight loss, and functional decline, further exacerbating impaired bone health. Protein-calorie malnutrition leads to decreased bone formation, impaired fracture healing, and loss of muscle mass [6]. On the other hand, chronic alcohol consumption disrupts calcium and vitamin D homeostasis and inhibits osteoblast function. [7] These factors are often considered in isolation; however, this case highlights the importance of recognizing coexisting behavioral or psychiatric factors.

A key feature of this case is the temporal relationship between the treatment of depression and subsequent clinical recovery. Following initiation of venlafaxine, on the 10th day of admission, the patient demonstrated sustained improvement in mood, accompanied by increased appetite, improved nutritional intake, weight gain, and enhanced engagement with rehabilitation. This was associated with significant functional recovery, including progression from wheelchair dependence to independent mobilization. Given that venlafaxine is not typically associated with appetite stimulation, the improvement in nutritional intake is more likely related to the resolution of depressive symptoms rather than a direct pharmacological effect.

Biochemical findings were interpreted in the context of the patient’s clinical presentation (Table 1). On admission, hyponatremia with relatively preserved hemoglobin was consistent with hypovolemic hyponatremia and hemoconcentration. Low urea and creatinine were attributed to reduced muscle mass in the context of sarcopenia. Hypoalbuminemia in a relatively young patient supported underlying malnutrition. Following nutritional rehabilitation and fluid repletion, hemoglobin decreased while sodium normalized, supporting an initial component of dehydration. Albumin improved from a nadir of 19 g/L to 30 g/L on discharge, in keeping with improving nutritional status. Calcium, borderline elevated on admission, normalized with rehydration, suggesting hemoconcentration. Overall, biochemical trends were consistent with a combination of dehydration, malnutrition, and metabolic adaptation during nutritional rehabilitation.

Radiological osteopenia and multiple low-impact fractures support underlying bone fragility. Although a formal bone mineral density assessment with dual-energy X-ray absorptiometry (DEXA) was not performed, the clinical presentation is consistent with reduced bone strength. While FRAX is not validated in younger populations, the elevated score in this case supports increased fracture risk in the presence of multiple clinical risk factors [9]. In older individuals, fragility fractures are commonly recognized as a manifestation of frailty [9]. In this patient, the fracture may represent a late manifestation of a reversible, frailty-like state associated with prolonged malnutrition and self-neglect.

This case highlights potential missed opportunities for earlier intervention. Progressive weight loss and reduced nutritional intake in the community may warrant earlier investigation, including assessment of bone health and coexisting behavioral or psychiatric factors. Early recognition may allow timely intervention and reduce the risk of progression to complications such as fragility fractures and functional decline [8].

Overall, this case emphasizes the importance of a holistic clinical approach in patients presenting with unexplained fragility fractures, particularly in younger individuals. Consideration of nutritional, behavioral, and psychiatric factors is essential to ensure comprehensive management and optimize outcomes.

## Conclusions

Fragility fractures in younger individuals should prompt evaluation for underlying systemic and behavioural factors. This case highlights the association between depression, self-neglect, malnutrition, and bone fragility. It also emphasises the importance of a multidisciplinary approach in the evaluation and management of this patient population. Early recognition of depression, substance misuse, and malnutrition may facilitate timely intervention, reduce progression to significant morbidity, and potentially decrease long-term healthcare burden.

## References

[1] Anastasopoulou C, Barnett MJ, Rodrigues Silva Sombra L, Garla VV (2025). Osteoporosis in males. StatPearls [Internet].

[2] Formosa MM, Christou MA, Mäkitie O (2024). Bone fragility and osteoporosis in children and young adults. J Endocrinol Invest.

[3] Kris-Etherton PM, Petersen KS, Hibbeln JR (2021). Nutrition and behavioral health disorders: depression and anxiety. Nutr Rev.

[4] Johnson JT, Hussain MA, Cherian KE, Kapoor N, Paul TV (2022). Chronic alcohol consumption and its impact on bone and metabolic health - a narrative review. Indian J Endocrinol Metab.

[5] Bell JJ, Geirsdottir OG, Johansen A (2025). The SIMPLER nutrition pathway for fragility fractures: a quality improvement initiative. Nutrients.

[6] Meesters DM, Wijnands KA, Brink PR, Poeze M (2018). Malnutrition and fracture healing: are specific deficiencies in amino acids important in nonunion development?. Nutrients.

[7] Eby JM, Sharieh F, Callaci JJ (2020). Impact of alcohol on bone health, homeostasis and fracture repair. Curr Pathobiol Rep.

[8] Qiu L, Yang Q, Sun N (2018). Association between depression and the risk for fracture: a meta-analysis and systematic review. BMC Psychiatry.

[9] Nuti R, Brandi ML, Checchia G (2019). Guidelines for the management of osteoporosis and fragility fractures. Intern Emerg Med.

